# User Attitudes Toward Research and Development of Technologies: Perspectives From Three Generations

**DOI:** 10.1093/geroni/igaa057.2990

**Published:** 2020-12-16

**Authors:** Steven Schmidt, Sofi Fristedt, Charlotte Löfqvist, Susanne Iwarsson

**Affiliations:** 1 Lund University, Lund, Skane Lan, Sweden; 2 Jonkoping University, Lund, Jonkopings Lan, Sweden

## Abstract

New technologies are being touted as solutions to many societal challenges not least of which are ageing and health. However, the rapid development of new technologies is proceeding with little input from older adults. This presentation highlights the perceptions and attitudes of three age cohorts related to the continuous technological advancement of products intended to support active and healthy aging. Participants were 30-39 (n= 639), 50-59 (n=703), 70-79 (n=779) years-old randomly sampled from the Swedish population registry. Results showed both similarities and difference across generations. For example, 24%-35% of older adults would like to use home monitoring devices (e.g. fall sensors, smart home devices) to support active and healthy aging, compared to 35%-56% of younger groups. More than 82% of all groups highlighted the importance of involving intended users in the development process. Results can be used to support the needs and desires of current older adults and future generations.

